# Microwaves and Ultrasound as Emerging Techniques for Lignocellulosic Materials

**DOI:** 10.3390/ma16237351

**Published:** 2023-11-25

**Authors:** Ana Fernandes, Luísa Cruz-Lopes, Bruno Esteves, Dmitry V. Evtuguin

**Affiliations:** 1 CICECO—Aveiro Institute of Materials, 3810-193 Aveiro, Portugal; ana.augusta@ua.pt (A.F.); dmitrye@ua.pt (D.V.E.); 2 Department of Environmental Engineering, Polytechnic Institute of Viseu, Av. Cor. José Maria Vale de Andrade, 3504-510 Viseu, Portugal; 3 Centre for Natural Resources, Environment and Society-CERNAS-IPV Research Centre, Av. Cor. José Maria Vale de Andrade, 3504-510 Viseu, Portugal; bruno@estgv.ipv.pt; 4 Department of Wood Engineering, Polytechnic Institute of Viseu, Av. Cor. José Maria Vale de Andrade, 3504-510 Viseu, Portugal; 5 Department of Chemistry, University of Aveiro, 3810-193 Aveiro, Portugal

**Keywords:** microwaves, ultrasound, lignocellulosic biomass, pretreatments, sonochemistry

## Abstract

Currently, in the context of biorefinery and bioeconomy, lignocellulosic biomass is increasingly used to produce biofuels, biochemicals and other value-added products. Microwaves and ultrasound are emerging techniques that enable efficient and environmentally sustainable routes in the transformation of lignocellulosic biomass. This review presents some of the most important works published in the last few years on the application of microwaves and/or ultrasound in lignocellulosic materials pretreatment and can be used as a starting point for research into this theme. This review is divided into four parts. In Part I, the theoretical fundamentals of microwave and ultrasound treatments are reviewed. Dielectric constants for biomass, factors that influence pretreatment, are some of the subjects addressed. In Part II, the effects that these techniques have on lignocellulosic biomass (on the size and surface area of the particle; on the content of lignin, hemicellulose and cellulose; on the crystallinity index of cellulose; on the effect of solubilization of organic matter; on hydrolysis and reduction of sugars) are discussed. In Part III, emphasis is given to the contribution of microwaves and ultrasound in obtaining value-added products. In this context, several examples of liquefaction and extraction procedures are presented. Part IV describes examples of performing sonocatalysis on lignocellulosic biomass to obtain value-added products, such as furfural, whose production is significantly reduced by ultrasound treatment.

## 1. Introduction

Lignocellulosic biomass is the most abundant renewable resource in the world. The availability of non-food biomass is estimated to be (170–200) × 10^9^ tons per day [1]. In the current context of biorefinery and bioeconomy, lignocellulosic biomass is increasingly used to obtain various alternative products to those of petroleum origin, namely biofuels, biochemicals, and other value-added products. Lignocellulosic biomass has a compact and robust structure that has been developed to acquire natural resistance in the cell wall and to protect itself from external physical or climatic actions and microbiologic attacks. This resistance is also called biomass recalcitrance [2,3,4,5]. Due to recalcitrance, lignocellulosic biomass must be pretreated before being chemically or biologically processed in order to obtain new bio-based products or biofuels. The goal of these pretreatments is to deconstruct the compact and recalcitrant structure of lignocellulosic biomass [3,5,6,7].

Pretreatments can be subdivided into four categories, depending on the approach: (i) physical: mechanical extrusion, grinding, microwave, ultrasound, pyrolysis, and pulsed electric field; (ii) chemical: acid, alkaline, ozonolysis, organosolv, ionic liquids, and deep eutectic solvents; (iii) physico-chemical: steam explosion, liquid hot water, wet oxidation, pretreatment with sulfite to recover lignocellulosic recalcitrance (SPORL), carbon dioxide explosion, and ammonia fiber explosion (AFEX); or (iv) biological: enzymatic, microbial, and fungal [3,6,8,9,10,11,12,13]. Depending on the type of biomass, the most appropriate pretreatment is selected. Often, it is necessary to perform a hybrid pretreatment—that is, a pretreatment that results from the combination between different types of pretreatments [8]. Each pretreatment has its advantages and drawbacks. Numerous studies have been published that describe, in detail, the specific drawbacks of each of the referred pretreatments (physical, chemical, physico-chemical, or biological) [2,13,14,15,16]. It should be noted that despite the large number of publications on the effect of different type of pre-treatments, when applied to lignocellulosic biomass, no single pretreatment was found to be superior in all respects [15,16]. This would also not be possible because, as will be discussed, the results of pretreatment vary from biomass to biomass.

Currently, within the scope of the concepts of biorefinery and circular bioeconomy, new pretreatment routes for lignocellulosic biomass have been developed in accordance with the principles of green Chemistry [14,15]. In this context, processes and routes are sought that simultaneously comply with four requirements: (i) do not consume too much energy; (ii) do not use toxic or dangerous solvents; (iii) minimize the amount of waste and be economically profitable [14,15]. In the search for approaches that meet these requirements, many studies on lignocellulosic biomass pretreatments have been published. Recent publications mention the following emerging techniques for the pretreatment of lignocellulosic biomass: ultrasound, microwaves, electron beams, gamma rays, high pressure homogenization, high-hydrostatic-pressure treatment, and pulsed electric field [15,17,18].

This work focuses on the application of microwaves and ultrasound as emerging techniques for lignocellulosic material pretreatment. Still, there are many challenges in using these technologies on an industrial scale, although ultrasound has already been used in the food industry for several years [19]. The objective was to review a set of studies and conclusions on the application of microwaves and/or ultrasound in the treatment of lignocellulosic materials that can be used as a guide for the research of this subject. Accordingly, this review includes: (1) effect of dielectric constants, dielectric losses, tangents of electrical losses for various lignocellulosic biomasses on the depths of penetration of microwaves (for example, as reported for seeds of karanja [20]); (2) microwave-assisted liquefactions via ultrasonic or hybrid methods (assisted simultaneously by microwaves and ultrasound) carried out with lignocellulosic biomass for the preparation of value-added products. For example, microwave-assisted liquefaction of bamboo sawdust to obtain a polyol for subsequent preparation of polyurethane foams, which was carried out in 8 min and with a yield of 78% [21]; (3) microwave-assisted, ultrasonic, or hybrid extractions performed with lignocellulosic biomass for the extraction of phenolic compounds. Thus, microwave-assisted extraction of phenolic compounds from coriander leaves was carried out in 4 min [22]; (4) sonocatalysis reactions performed on lignocellulosic biomass for the preparation of value-added products. Several examples of the synthesis of 5-hydroxymethylfurfural (5-HMF) are cited for this purpose, one of them from banana peels [23].

This review paper is structured in four parts. In Part I, the basic principles of microwave radiation and ultrasound treatment are presented. With regard to microwave technology, some of the topics exposed are as follows: (i) an introduction to its basic principles is given; (ii) a distinction is made between conventional heating and microwave heating; (iii) description of the heating mechanisms using microwaves; (iv) the advantages and disadvantages of this type of heating are explored; (v) exploration of the behavior of lignocellulosic biomass when subjected to microwave radiation; (vi) the factors that influence the dielectric parameters of the biomass are indicated; and (vii) discussion of issues to consider in microwave pretreatment for lignocellulosic biomass. Regarding ultrasound, (i) the phenomenon of cavitation is described, (ii) the physical and chemical effects of cavitation on lignocellulosic biomass are disclosed, and (iii) the parameters to be considered in an ultrasonic treatment are discussed. Part II presents the main effects of microwave radiation and ultrasound treatment on lignocellulosic biomass, and some examples of stand-alone or combined pretreatments are revealed in which these effects have been evaluated. In selecting these examples, the criteria were to present, in addition to autonomous pretreatments, combined ones which use microwaves or ultrasound and to select pretreatments from different categories (which use acids, alkaline solutions, ionic liquids, ammonia, avoiding steam explosion, among others) to provide an overview of the applicability of these two emerging technologies on different routes. In this part, the effects that microwaves and ultrasound have on lignocellulosic biomass are discussed, namely: (i) on the size and surface area of the biomass particles; (ii) lignin, hemicellulose, and cellulose content; (iii) on the cellulose crystallinity index; (iv) the effect of solubilization of organic matter and the hydrolysis and reduction of sugars. In Part III, emphasis was placed on the contribution of microwaves and ultrasound techniques to the production of value-added products from lignocellulosic biomass. In this part, several examples of liquefaction (under moderate conditions, pressure, and temperature) and solvent extractions in which microwave and ultrasound treatments are presented. Only examples of extractions that have the common objective of obtaining phenolic compounds from lignocellulosic biomass are presented. Finally, in Part IV, the main sonocatalysis approaches involved in obtaining value-added products from lignocellulosic biomass are reviewed. The examples presented are laboratory bench studies that were later implemented on a pilot scale and then on an industrial scale.

## 2. Basic Principles of Microwave and Ultrasound Treatments (Part I)

### 2.1. Microwaves Radiation

Microwave radiation is non-ionizing radiation that lie between radio waves and infrared on the electromagnetic spectrum. Microwaves, like all electromagnetic waves, are composed of two fields perpendicular to each other, the electric and magnetic fields, which oscillate in the frequency range from 300 GHz to 300 MHz, corresponding to wavelengths from 1 mm to 1m, respectively [24,25]. This radiation is classified as non-ionizing because it does not have enough energy to remove electrons from the molecules or atoms on which it acts; it can only increase their kinetic energy, which translates into an increase in temperature [26].

Microwave radiation used at the industrial level has a frequency of 915 MHz, which allows more uniform heating and a transformation efficiency into heat of 85% [24,26]. Conventional microwave kitchen ovens typically use a frequency of 2.45 GHz, and their efficiency is 50% [24,26]. Most microwave reactors used for chemical synthesis also operate at 2.45 GHz [24]. Microwave photons do not ionize because they have low energy, unlike ionizing γ-ray or x-ray photons. Microwave photon frequencies of 915 MHz and 2.45 Hz correspond to energy values of 0.09 cal/mol and 0.23 cal/mol, respectively [26]. Typical photon energy from microwave radiation is ca. 0.03 kcal/mol, and the energy of chemical bonds ranges from 20 to 50 kcal/mol. [8]. Accordingly, these energy values of different orders show why microwave radiation is non-ionizing.

From a historical point of view, it is important to note that microwaves began to be used in industrial applications around the year 1980 [8,27]. Engineer Percy L. Spencer discovered in 1949 that microwave radiation can heat materials [8,14], but the first theory about the interaction of microwaves with matter was elaborated around the year 1954 by Von Hippel [8,14].

#### 2.1.1. Conventional Heating and Microwave Heating

**Figure 1:**
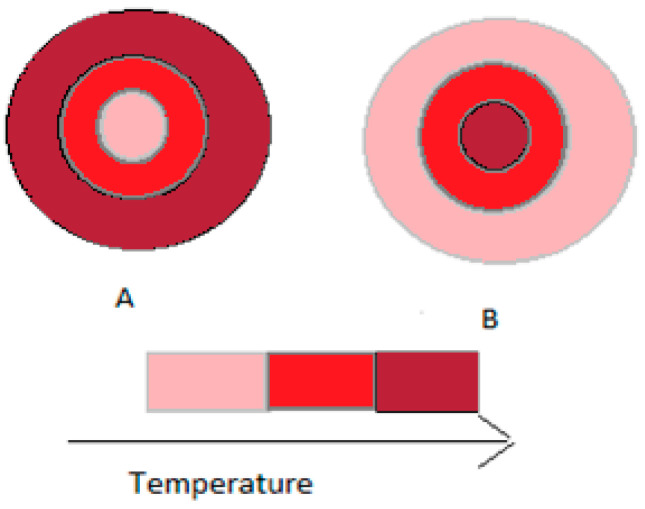
Representation of a conventional heating profile (**A**) and microwave-heating profile (**B**). Adapted from [28].

There are several differences between conventional and microwave heating. In conventional heating, the wall of the container is heated first, and only then is the material inside it heated (according to profile A, from Figure 1). In microwave heating, the process is reversed, first heating the inside and then the outside of the material (according to profile B, Figure 1).

The main differences between conventional and microwave heating in auto-hydrolysis treatment have been reported before [29,30]. The work conducted by Dávila et al. [29] highlighted the environmental sustainability of the microwave-assisted auto-hydrolysis method in the valorization of vine residues. In this method, a lower production of oligosaccharides was observed, as was an energy consumption only 28.8% of that required for conventional thermal treatment. Additionally, conventional hydrothermal treatment consumed 2.1 to 2.8 times more energy than microwave hydrothermal treatment, highlighting the energy efficiency of microwave-assisted auto-hydrolysis technology in the transformation of Paulownia elongata [30]. These results emphasize that the use of microwaves in the hydrolysis process is a sustainable and efficient alternative for this plant species.

These results are promising and encourage further research in this area, aiming to make the most of natural resources in a responsible manner. There are several studies that mention the advantages of heating lignocellulosic biomass with microwaves compared to conventional heating [8,15,25,28,29,30]. Table 1 summarizes some of these advantages.

However, the heating of lignocellulosic biomass via microwave incidence also has disadvantages. The existence of materials that have low absorption, the presence of materials whose dielectric properties change with temperature, and the occurrence of heterogeneous materials (in composition, shape and/or size range) are some of these disadvantages. The existence of heterogeneous materials has as a consequence the differentiated absorption of heat, and a local overheating and the formation of the so-called “hot spots” can occur [8,14,25,26]. This phenomenon of the formation of “hot spots” can be minimized by increasing the size of the cavity, working at a higher frequency, or by coupling an agitator/turntable [26].

#### 2.1.2. Microwave Heating Mechanisms

**Figure 2:**
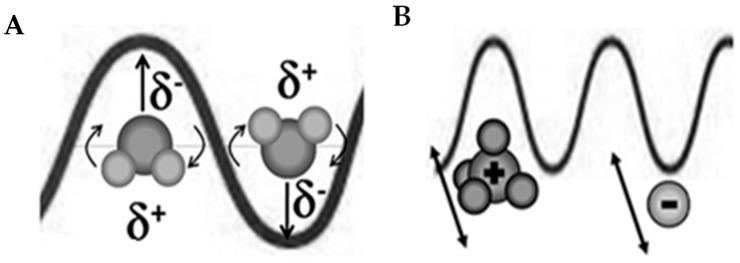
Illustration of microwave heating mechanisms: (**A**) dipolar rotation mechanism for polar molecules; (**B**) ionic driving mechanism for ions. Adapted from [31] with the permission of Scielo 2015.

Microwave heating is a non-contact energy transfer process that can be accomplished via two distinct mechanisms: dipole rotation and ion conduction (Figure 2) [8,14,24].

In the case of polar molecules, these molecules have a tendency to orient according to the alternating electric field of the microwave radiation, and this generates heat due to rotation, as it causes friction and collision between the molecules [8,14,24]. This mechanism is called dipole rotation (Figure 2A). This mechanism occurs in polar molecules that have permanent dipoles but also in molecules with induced dipoles [24]. In the case of ions, the interaction with the alternating electric field causes these charged particles to move, constantly changing direction; that is, they move back and forth, which causes a local increase in temperature due to friction and collision between the ions [8,14,24]. This mechanism is called ion conduction heating (Figure 2B). This ion conduction mechanism has more influence on heat generation than the dipole rotation mechanism [24]. It should be pointed out that the electric field is called alternating because its direction is constantly changing. For a frequency of 2.45 GHz, the direction of the electric field oscillates about 4.9 billion times per second [32].

#### 2.1.3. Behavior of Materials in Relation to Microwave Radiation

There are three parameters for evaluating the behavior of materials in the face of microwave radiation: the dielectric constant ($\varepsilon^{\prime })$, the dielectric loss ($\varepsilon^{\prime \prime }$), and the dielectric loss tangent (tan $=\frac{\varepsilon^{\prime }}{\varepsilon^{\prime \prime }}$).

The dielectric constant ($\varepsilon^{\prime })$, relates to the ability of molecules to be polarized by an electric field. In other words, it is the ability of molecules to store electromagnetic energy [2,24,33]. This quantity depends on the molecular mass and geometry of the molecule [8].

Dielectric loss ($\varepsilon^{\prime \prime }$), measures the ability of a material to convert energy into heat [2,24,33]. The lower the dielectric loss for a material, the lower its ability to absorb microwaves [25,27]. $\varepsilon^{\prime \prime }$ decreases with increasing temperature [8,25].The fact that the electrical loss decreases with temperature makes this parameter possible to be modified by changing the temperature [8].

The dielectric loss tangent (tan δ) results from the mathematical relationship between these properties and so is a dimensionless parameter. This value reveals the ability of a material to be heated by microwave [8,24]. If this parameter is null it means that this material does not heat up with the incidence of microwave radiation [8,25,34].

According to the behavior of the materials in the face of microwave incidence, the materials can be classified into three categories: dielectric, conductive and non-conductive [8,26,34]. Table 2 presents the characteristics, the dielectric loss tangent, and examples for conductive, non-conductive, and dielectric materials.

According to the literature, lignocellulosic biomass can be classified as a low-loss dielectric material, which—in other words—means that biomass absorbs microwave radiation but with some difficulties [24,33].

Another important parameter which reveals the behavior of a material affected by microwave radiation is the depth of penetration, D_p_. This parameter estimates how deep microwave radiation reaches into a given material and can be predicted using the following expression [33].

$$D_{p}=\frac{\lambda_{0}}{2\pi (2\varepsilon^{\prime }{)}^{0.5}}{\left\{[1+({tan\;\delta )}^{2}\right]}^{0.5}-1\;{\}}^{-0.5}\;$$

(*λ*_0_—is microwave wavelength in free space).

For water, the penetration depth is 1.4 cm at a temperature of 25 °C, but increasing the temperature to 90 °C increases it to 5.7 cm (maintaining a frequency of 2.45 GHz) [25].

#### 2.1.4. Behavior of lignocellulosic Biomass in Relation to Microwave Radiation

The behavior of lignocellulosic biomass against microwave radiation depends, as previously mentioned, on the effective parameters: $\varepsilon^{\prime },\;\varepsilon^{\prime \prime }$, tan δ, and D_p_. It is therefore essential to know the values of these parameters for lignocellulosic biomass when applying a microwave treatment. In the literature, there are examples of evaluation of these parameters for the various biomass, for examples: palm bark and fibers [32]; empty fruit bunches [35]; tropical wood [36]; banana fibers with polyurethane [37]; pinewood blocks [34]; pinewood and arabica coffee [38]; hay [39]; and karanja seeds [20]. Table 3 shows the dielectric constants, dielectric losses, dielectric loss tangents, and depths of microwave penetration for various biomasses.

As shown by the examples in Table 3, the dielectric properties vary substantially according to the type of biomass. These dielectric parameters are not constant and depend on temperature [27]. Regarding the loss tangents of the various biomasses presented in Table 3, it is necessary to highlight that of karanja seeds [20]. These seeds exhibit a value of 1.3 for dielectric loss tangent (tanδ) (at 2.45 GHz). This value is the highest known loss tangent value for lignocellulosic biomass. This makes this biomass ideal to be used in microwave pyrolysis for biodiesel production, moreover because these seeds are not edible.

The values of the dielectric constants of a biomass also depend on its humidity. The values of the dielectric parameters relative to the empty fruit bunch, in Table 3, are for 18% humidity [35]. However, if the moisture of the empty fruit cluster is 64% instead of 18%, the dielectric constant and the dielectric loss tangent increase from 6.4 to 57.4 and from 1.9 to 18.6, respectively.

There are several studies on the dielectric parameters of biomass. In one study, Salema et al. measured the dielectric properties of five different agricultural and forest residues (palm bark, empty fruit cluster, coconut husk, rice husk, and wood sawdust) from room temperature to approximately 700 °C [33]. This study mentions that the dielectric constants decrease slightly during the drying phase (from 24 to 200 °C); during pyrolysis, the dielectric constants continue to decrease (from 200 to 450 °C), but after 450 °C, the dielectric constants increase significantly. These researchers concluded that the dielectric constants depend on the type of biomass and vary during drying and pyrolysis but that they vary nonlinearly with temperature.

In another study, the parameters $\varepsilon^{\prime }\;$and tan *δ* were measured for different lignocellulosic fibers (residual lemon, medlar, palm, and olive leaves) for a frequency range from 10 Hz to 8 MHz, and the authors concluded that with an increase in the frequency of the microwaves, the dielectric constant decreases [36]. However, the loss tangent decreases until it reaches a minimum and then remains constant [40]. It was verified that all fibers exhibited the same behavior. These dielectric parameters were measured for lignocellulosic fibers under the same conditions: at room temperature with a frequency of 10 Hz to 8 MHz for peak voltage 1 V and from 10 Hz to 100 KHz for peak voltage 5 V. It was concluded that these dielectric parameters depend in a notable way on the frequency. The dielectric materials were subdivided into four categories as follows: homogeneous (when the electrical properties are independent of position); dispersive (when the electrical properties depend on the frequency variations of the electric field); isotropic (when they are not affected by the direction of the applied electric field), and linear (when they are independent of the strength of the applied electric field [40]. This allowed classification of lignocellulosic biomass as a dispersive material, with a good degree of homogeneity and linearity.

In the treatment of lignocellulosic biomass, solvents are normally used. A group of researchers studied the incidence of microwaves in water, aqueous acidic, and alkaline media and in an ethanol–water mixture and concluded that the best solvent for the absorption of microwave radiation is water [41]. In fact, it is known that the presence of water inside the materials facilitates their heating and that humidity influences the dielectric properties, as it influences the D_p_ [8].

It is also important to know the dielectric parameters of the solvents used in the treatments assisted by microwaves: $\varepsilon^{\prime },\;\varepsilon^{\prime \prime },$ and tan *δ*. These dielectric parameters are summarized for various solvents in Table 4.

The higher the tan *δ*, the more polar the solvent and the more easily it heats up due to the action of microwaves. The analysis of Table 4 shows that water is the best microwave absorber (as previously mentioned). In microwave-assisted pretreatments usually applied to lignocellulosic biomass the most commonly used solvents are water, aqueous acidic or basic solutions, deep eutectic solvents, and ionic liquids (as will be seen later) [42]. For these solvents, before using them, it is advisable to know their dielectric parameters.

#### 2.1.5. Microwave Absorbing Materials Addition to Lignocellulosic Biomass

Since lignocellulosic biomass does not absorb microwave radiation well [33], microwave-absorbing materials—that is, materials with high tan *δ*—should be added to the biomass; for example, before pyrolysis, the microwave absorbers are added [24]. Microwave-absorbing materials are carbon-based solid materials and metal oxides. In the category of metal oxides, the most used are: CuO, MgO, Fe_2_O_3_, Al_2_ O_3_, and SiO_2,_ and in the category of carbon-based solid materials, the most common are coal, activated carbon, coke, graphite, and silica carbide (SiC) [24,31,39,40].

Although microwave-assisted pyrolysis is not the subject of this review, for a better understanding of the behavior of lignocellulosic biomass when subjected to microwaves, the advantages and disadvantages of these two types of microwave-absorbing materials are presented below. Microwave-assisted pyrolysis (MAP) has proven to be an effective method of shortening pyrolysis reaction times and improving the quality of value-added products from several types of raw materials, eliminating the need for grinding [43]. Several reviews have been conducted in recent years on microwave-assisted pyrolysis [43,44,45,46,47,48,49,50]. There are several differences between conventional and microwave-assisted pyrolysis. Microwave-assisted pyrolysis has uniform heating—the whole material is heated simultaneously—while in conventional pyrolysis, there is superficial heating and then a transfer of energy via convection and/or conduction [49]. Furthermore, the heating in MAP is rapid and efficient and is more precise and controlled since it is possible to stop the heating immediately by turning off the power [49]. The most important operational parameters influencing product yield in MAP have been stated to be microwave power, temperature, addition and concentration of microwave absorbers, initial moisture content, and the flow rate/residence time of the initial sweep gas [44]. The addition of microwave absorbers can lead to enhancement of the pyrolysis reaction temperature at relatively low microwave power. There are several microwave absorbers used for lignocellulosic biomass—for example, silicon carbide used in MAP of pine wood sawdust [51] or MgCl_2_ and Na_2_HPO_4_ in corn stover pellets [52]. Sometimes, pyrolysis char is also used as a microwave-absorber [53,54,55]. With regard to carbon-based solid materials, the researchers point to three advantages with respect to microwave-assisted heating, namely: (1) the increased absorption capacity of microwaves of bulk materials; (2) increased heat transmission to surrounding materials; and (3) increased heating rate at low microwave powers [25]. Carbon-based solids are good microwave absorbers, as they have high D_p_ values when compared to metals. Activated carbon, for example, has a D_p_ of 0.7 to 3.43 cm, and silver has a D_p_ of 1.3 μm (values for frequency 2.45 GHz and room temperature [25]). As for the disadvantages of adding carbon-based solid materials, they can influence yields and alter the desired product yields [24].

Adding metal oxides to lignocellulosic biomass to make it more microwave-absorbent also has several advantages. At least three advantages are mentioned in the literature, namely: (1) the increase in the absorption capacity of microwaves; (2) increasing the rate of warming, and (3) “improving the devolatilization” of biomass [24].

Comparing carbon-based solids to metal oxides with materials, researchers report that metal oxides can affect the quality of the product obtained by pyrolysis [24], and carbon-based solids appear to be preferable because they mixed better and more evenly with the biomass [56].

#### 2.1.6. Factors to Consider in a Microwave Pretreatment for Lignocellulosic Biomass

As was evidenced in the previous sections of this review, there are several factors that affect the heating of lignocellulosic biomass when subjected to microwaves and these factors should be studied in detail beforehand. Prior knowledge of dielectric properties ($\varepsilon^{\prime },\;\varepsilon^{\prime \prime },$tan *δ*, and D_p_) of the lignocellulosic biomass is important not only for a better understanding of the microwave heating process but also for a proper sizing of the necessary equipment [33]. A recent review [3] summarizes seven factors that affect the heating of lignocellulosic biomass by microwaves: (1) the dynamics of dipole biomass molecules; (2) the composition and size of the biomass; (3) the induction current of magnetic materials present in the biomass and the ionic conduction of electrolytes; (4) reaction time (residence time) and heating rate; (5) the moisture content of the biomass; (6) the power of the microwave; and (7) the depth of penetration. Therefore, in the selection of a microwave pretreatment, or in the sizing of a microwave equipment, these variables must be considered.

Since several factors influence the behavior of lignocellulosic biomass, when subjected to microwave radiation, and these factors being interconnected with each other, it becomes difficult to find the best conditions for a given pretreatment employing microwaves. To overcome this barrier, a computational simulation was recently developed on the Comsol Multiphysics platform, using Maxwell’s mathematical equations and the heat transfer equation to simulate microwave heating for three types of lignocellulosic biomass: sugarcane bagasse, palm oil, and green algae [25]. The goal was to find the best conditions for microwave pretreatment for these three types of biomass, and they concluded that these conditions depend on temperature, humidity (from 20 to 80%), volume (from 10^−5^ to 100 × 10^−5^ m^3^), and the shape of these samples (cylindrical or spherical). This work allowed us to reach the following conclusions: (i) the selection of the microwave power is fundamental to finding the best conditions (temperature, humidity, volume, and shape of the sample); (ii) since there is a homogeneous temperature distribution profile inside the sample, the sample size and the penetration depth (D_p_) must have dimensions of the same order of magnitude; (iii) materials with high values of dielectric constants (ε′) and dielectric losses (ε″) will have low penetration depth values (D_p_); (iv) the distribution of the electric field depends on the geometry of the sample, the humidity, and also the type of biomass; and (v) the power absorbed by the sample increases with its volume but decreases with the quotient of its surface area/volume [25].

#### 2.1.7. The Reasons Justifying Microwave Absorption and Lignocellulosic Biomass Recalcitrance

As already discussed, lignocellulosic biomass is a dielectric material, which absorbs microwaves. The main causes of the absorption of microwaves by lignocellulosic biomass are the presence of water and the polarity of the macromolecules that constitute the biomass (cellulose, hemicellulose, and lignin). With regard to polarity, it is the polarity of the macromolecules from the biomass that provides its heating when microwave irradiation reaches these macromolecules (dipole rotation mechanism as discussed before).

It is important to emphasize that the polarity of the constituent macromolecules of biomass is due, in part, to hydroxyl groups (-OH) [8,14]. These OH groups establish hydrogen bonds within the polymeric molecules of the biomass and between these polymers (intra-polymer and inter-polymer bonds, respectively) [5]. A set of all these intra-polymer and inter-polymer hydrogen bonds and cross-links between macromolecules gives robustness to lignocellulosic biomass and makes it recalcitrant and very difficult to deconstruct (Table 5).

Several factors related to the structural features and morphology of lignocellulosic biomass were referred as contributing to the recalcitrance of biomass: (i) the crystallinity of cellulose, the degree of polymerization, (ii) the size of the biomass particle, (iii) the size and volume of the pores in biomass tissues, (iv) the accessible surface area, and (v) the structural complexity of biomass components [5]. Before lignocellulosic biomass is used in the processing to produce bio-based products or biofuels, it has to be deconstructed, and this is the main function of any pretreatment (Part II). The deconstruction of lignocellulosic biomass by the incidence of microwave radiation is possible because the microwaves force the dipolar macromolecules of the biomass to align with the oscillating electric field, which results in the rupture of hydrogen bonds and the breakdown of cell walls [57].

### 2.2. Ultrasound and Two Categories of Ultrasound

Sounds in the sound spectrum can be classified, according to applied frequency (*f*), into three groups: infrasounds (*f* < 20 HZ), audible sounds (20 Hz < *f* < 20 kHz), and ultrasound (*f* > 20 kHz). In turn, ultrasound can be subdivided into two categories: (1) low- to medium-frequency waves (20–100 kHz), also called “power ultrasounds” and (2) high-frequency waves (3–10 MHz), also called “diagnostic ultrasounds”. Table 6 summarizes some of the main differences between the two categories of ultrasonics [27,58,59].

Regarding an ultrasound treatment applied to the pretreatment of lignocellulosic biomass, low- and medium-frequency ultrasound are commonly reported.

#### 2.2.1. Basic Principles of Cavitation

An ultrasound is a cyclic pressure wave consisting of compression and rarefaction zones (areas of low pressure) alternating in space and time. When an ultrasound (of low or medium frequency) propagates inside a liquid, the phenomenon of acoustic cavitation occurs. This phenomenon originates in a zone of rarefaction (or zone of negative pressure) that, when propagating inside a liquid, forces its particles to separate, thus generating cavities or bubbles. As the wave travels through the liquid, the bubbles grow for successive cycles until they reach an unstable size and then suffer a violent collapse [60,61,62,63].

**Figure 3:**
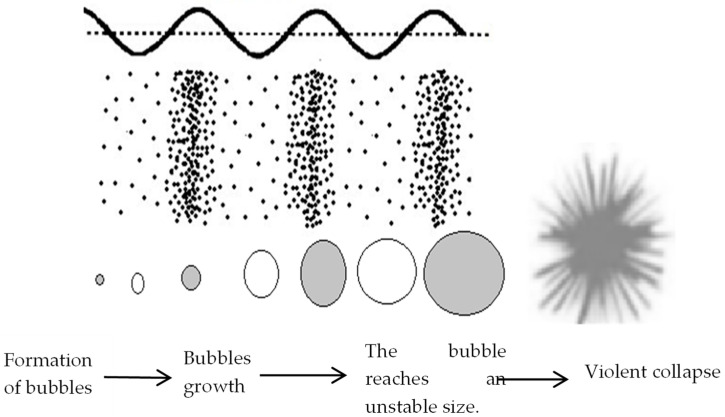
Illustration of cavitation phases: from blister formation to violent collapse. Adapted from [61] with permission from ACS Publications.

Briefly, the phenomenon of acoustic cavitation has three phases: (1) the formation of the bubble; (2) the rapid growth of the bubble during the successive alternating compression-rarefaction cycles until it reaches an unstable size; and (3) the violent collapse of the bubble inside the liquid (Figure 3).

In most situations, after the collapse of the bubble, new smaller bubbles result and the cavitation cycle repeats. During the collapse of the bubble, the temperature and pressure inside it can reach very high values. The literature suggests temperature values between 500 K to 15,000 K and pressure values from 100 atm to 5000 atm [3]. Researchers say the life cycle of a bubble can last only a few microseconds [2] and that the rate of warming can reach 10^10^ kelvins per second [62]. Bubble collapse is a violent phenomenon that causes points of high temperature and pressure called “hot spots”. It is these “hot spots” that are the theoretical foundation of any pretreatment using ultrasound [60].

From a historical point of view, the phenomenon of cavitation was discovered by Thomycroft and Bamby in 1895, but it was not in demand until the year 1917, when the first mathematical model of the phenomenon was realized and disseminated by Lord Rayleigh [62].

#### 2.2.2. Factors That Influence the Cavitation of Lignocellulosic Biomass

The phenomenon of cavitation, when applied to lignocellulosic biomass, is a complex phenomenon that depends on numerous factors, such as the physical properties of the solvent used in the pretreatment, viscosity, surface tension, and volatility [4,64]. The literature states that the phenomenon of cavitation occurs preferably in liquids with low volatility, medium viscosity, and high surface tension [64]. In addition to these factors, in a pretreatment with ultrasound, it is necessary to consider not only the frequency of the ultrasound, the sonication time, and the acoustic power of the ultrasound but also the geometry of the reactor (ultrasonic baths and probes are different) [11,59,61,64]. Temperature is also a factor that has an influence when applying an ultrasonic treatment to lignocellulosic biomass [59]. Thus, in aqueous solvents, cavitation is at its maximum at low temperatures [61].

In addition to the aforementioned factors, the effectiveness of an ultrasonic pretreatment also depends on the type of lignocellulosic biomass. Studies show that when different biomasses are applied for the same treatment, this leads to different experimental results [61]. For this reason, when optimizing a treatment route for a given biomass, this route is commonly suitable for that biomass only [61]. The same route applied to other biomass may prove to be ineffective.

Thus, briefly, it can be stated that the effectiveness of ultrasonic pretreatment depends on the following factors: the properties of the medium (solvent viscosity, surface tension, and volatility); the characteristics of ultrasound (frequency, sonication time and power); the operating temperature; and the type of biomass.

#### 2.2.3. Physical Effects and Chemical Effects of Ultrasound on Lignocellulosic Biomass

The ultrasound effects on lignocellulosic biomass are very diverse and complex but can be subdivided into two main groups: (i) the physical or mechanoacoustic effects and (ii) the chemical or sonochemical effects [2,61,62,64,65]. The physical effects during cavitation deal with the formation of strong shear forces and the creation of microjets [64,66]. Shear forces and microjets are the consequence of symmetrical cavitation (usually spherical) or asymmetric cavitation, respectively [64]. Microjets form when cavitation occurs on the surface of a solid particle larger than the bubble [61]. It is important to mention that microjets can reach projection speeds of hundreds of kilometers per hour [61].

As for the chemical effects of ultrasound, the formation of several radicals stands out [2,60,61,62]. The most important free radicals to consider are those that result from the ultrasonic decomposition of water, the hydroxyl radical (OH^●^) and the hydrogen radical (H^●^) being the most important [66]. Note that the decoupling of these OH^●^ and H^●^ radicals can, in turn, form water, or the two hydroxyl radicals can react with each other and form hydrogen peroxide [61,62]. However, the dominant species is the hydroxyl radical, as its reduction potential (+2.06 V) is higher than that of hydrogen peroxide (+1.78 V) [58]. In addition to these radicals, others can be formed depending on the solvent applied in the ultrasonic treatment. In the literature, eight techniques are listed for measuring the concentration of different radicals resulting from cavitation [58]. Although the formation of radicals is the most relevant chemical effect, within the bubbles generated in cavitation, high temperature and pressure can cause luminescence phenomena [61].

The shear forces and the formation of microjets at high speeds lead to the detachment and destruction of the chemical bonds between the macromolecules of the lignocellulosic biomass [4]. As for the oxidizing radicals resulting from cavitation, they trigger numerous chemical reactions, which promote the decomposition of the macromolecules that constitute the lignocellulosic biomass and which are catalyzed by ultrasound (Part IV exposes the main reactions triggered by ultrasound in the lignocellulosic biomass).

Still, regarding the effects of acoustic cavitation, it is noteworthy that the main general physicochemical effect is the promotion of mass and energy transfers and—as a consequence—an increase in the speed of the chemical reactions involved [4,67]. This is due to the fact that acoustic cavitation promotes localized increases in temperature and pressure in very short time intervals, which—in addition to the turbulence and intensity of the shear forces and microjets—can cause morphological changes in the lignocellulosic biomass as well as an increase in the speed of the chemical reactions involved [3]. As for changes in the lignocellulosic structure due to ultrasonic pretreatment, as examples, the breakdown of the α-*O*-4 and β-*O*-4 bonds of the lignin is reported [14,66] as is the rupture of ether bonds between hemicellulose and lignin [14].

## 3. Pretreatments with Microwave and/or Ultrasound (Part II)

### 3.1. The Types of Microwave and/or Ultrasound Pretreatments

The main microwave-assisted pretreatments for lignocellulosic biomass are those that use water, acidic or alkaline solutions, deep eutectic solvents, and ionic liquids as reaction media [42]. Ultrasound-assisted pretreatments are classified as autonomous, those who use water, acidic or alkaline solutions; ionic liquids; organic solvents; inorganic salts and enzymes; or TiO_2_ [42]. The main function of these pretreatments is to deconstruct lignocellulosic biomass and to give it new functional chemical groups to produce biofuels and bio-based products as an alternative to petroleum products.

### 3.2. Physical Effects and Chemical Effects of Microwave and Ultrasound on Lignocellulosic Biomass

As was already mentioned, when microwave radiation is applied to lignocellulosic biomass, the presence of water in the biomass makes it more absorbent [8,56]. Another factor that contributes to the absorption of microwaves is the existence of ions [8,56]. Microwave absorption can also be increased by adding absorbent materials (as seen in Section 2.1.5). The objective is that the biomass macromolecules (lignin, hemicellulose, and cellulose) undergo dipole rotation when subjected to microwaves, so there is a rupture of the bonds and the common lignocellulosic structure.

Similarly to microwave action, when biomass is subjected to ultrasound, there is the phenomenon of cavitation, which also leads to the same consequence, the rupture and disorder of the lignocellulosic structure. Thus, the macro changes that ultrasound or microwaves cause in lignocellulosic biomass are similar. These changes, however, are affected by five main factors to consider: (i) the biomass particle size and surface area; (ii) the effect on lignin, hemicellulose, and cellulose content; (iii) the effect on the cellulose crystallinity index; (iv) the effect on the solubilization of organic matter; (v) and the effect on hydrolysis and the reduction of sugars [57,60,68,69]. Below, some examples showing each of these effects are presented and discussed.

#### 3.2.1. Effect on Particle Size and Surface Area

The effects of biomass particle size in a microwave-assisted alkaline pretreatment of the herb extraction process were evaluated [70]. Thus, the particle size and the specific surface area were measured under four different conditions: (i) without any pretreatment; (ii) with an alkaline pretreatment; (iii) with water pretreatment, assisted by microwaves; and also (iv) with alkaline pretreatment, assisted by microwaves. In this study, the alkaline pretreatment, assisted by microwaves, was the most effective in reducing particle size (from 197.0 to 163.5 μm) and increasing specific surface area from 0.38 to 0.63 m^2^ g^−1^ (Table 7). Thus, alkaline pretreatment assisted by microwaves, was the best route to increasing the surface area. The objective of this study was to find a route leading to the largest surface area increasing the accessibility of anaerobic biodigestion [70].

Another group of researchers studied the conversion of microcrystalline cellulose with various particle sizes, treating it with NaOH with and without applying microwaves. They concluded that when using only the alkaline pretreatment, the surface area of the particles increased by 56%, but if this pretreatment was assisted by microwaves (P = 800 W for 20 min), the increase was 75% [71].

In the study by Khanal et al. [72], the ground corn paste was subjected to ultrasound (US) to increase the extent of liquefaction in ethanol production. In this work, the corn paste samples were submitted to pretreatment with ultrasound, and a 20-fold increase in particle size in relation to the control experiment was registered.

González-Fernández et al. conducted a study in which they applied ultrasound, frequency 20 Hz, of five different energy levels for 15 min to *Scenedesmus microalgae* and analyzed the particle size distribution [73]. For untreated biomass, the first peak in the size distribution profile was 7.4 μm, while with pretreatment with ultrasound, this peak changed to 5.1 μm (Table 7).

Recently, microwave expansion pretreatment was used to extract hemicellulose from hemp stalk [74]. This work consisted of two parts: in the first part, the microwave expansion (MEP) was conducted, and in the second, the microwave-assisted alkaline extraction process (MAAE) was applied. To perform the MEP, the hemp stalks were peeled and placed with an alkaline solution of ammonium bicarbonate (NH_4_HCO_3_) in a microwave and only then, the MAAE was carried out. The results showed that the specific surface area increased from 1.30 to 1.85 m^2^ g^−1^ without MEP and with MEP, respectively (Table 7). In this work, the average pore size increased from 10.66 × 10^−3^ to 13.08 × 10^−3^ nm. The success of this work is due to the MEP method, in which the ammonium bicarbonate blowing agent decomposed, due to microwave heating, into a mixture of gases (NH_3_, CO_2_, and H_2_O). This mixture of gases caused an instantaneous increase in pressure, which contributed to the rupture of the cell wall [74]. These results were achieved with only 3 min of microwave incidence (P = 1100 W).

Another groundbreaking work deals with an innovative route of fractional precipitation of cavitation by means of negative-pressure-generated ultrasound [75]. This method was developed with the aim of purifying (+)-dihydromyricetin. This compound is an important bioactive flavonoid that can be obtained from the medicinal plants *Hovenia dulcis* and *Ampelopsis grossedentata*. According to established practice, fractional precipitation is a simple process based on the difference in solubility. Such a process has been used for this purpose since 2008, but conventionally, it takes 32 h. In this work, the researchers were able to achieve a yield of 97.56% in one minute using ultrasound. Particle size without treatment and with treatment fell from 14.802 μm (at zero pressure) to 3.719 μm at a negative pressure of 200 mm Hg (Table 7). It was demonstrated that the addition of ultrasound to the fractional precipitation increased the production yield of that flavonoid to practically 100%, and in addition, it extraordinarily decreased the required extraction time.

The application of a microwave- or ultrasound-assisted pretreatment to lignocellulosic biomass results in its fragmentation, which translates into a decrease in particle size and an increase in surface area [57,60,68].

**Table 7:** Effect of microwave radiation and ultrasound on the size and surface area of the particle.

Biomass	Pretreatment	Particle Size (μm)	Specific Surface Area (m^2^ g^−1^)	References
Residues from the herb extraction process	No treatment	197.0	0.38	[70]
	Alkaline (NaOH)	187.5	0.44	
	Water and MW	179.8	0.51	
	Alkaline (NaOH) + MW	163.5	0.63	
Hemp stalk	No treatment	……….	1.30	[74]
	With MEP ^1^	…….	1.85	
Microalgae *Scenedesmus*	No treatment	7.4	N. d.	
	US	5.1	N.d.	[73]
*Hovenia dulcis* and *Ampelopsis grossedentata*	No treatment	14.802	N.d.	[75]
	Negative pressure and US ^2^	3.719	N.d.	

^1^ MEP—microwave expansion pretreatment. ^2^ Pretreatment with ultrasound and negative pressure fractional precipitation methods. N.d.—not determined.

#### 3.2.2. Effect on Lignin, Hemicellulose, and Cellulose Content

Several studies have shown that the use of microwaves or ultrasound significantly alters the content of the three main constituents of lignocellulosic biomass (cellulose, hemicellulose, and lignin). Thus, sugarcane bagasse was treated under acidic conditions employing a microwave (MW) irradiation [76]. This acid-catalyzed pretreatment was performed for three different temperatures (130 °C, 160 °C, and 190 °C) and for two heating times (5 min and 10 min). It was observed via scanning electron microscopy (SEM) that at 190 °C, within 10 min of MW incidence, the lignocellulosic structure was completely destroyed. Hence, even a 5 min MW pretreatment at 190 °C is sufficient to eliminate easy hydrolysable polysaccharides. Under these conditions, the cellulose, hemicellulose, and lignin contents were evaluated, and it was verified that almost all the hemicelluloses were removed (from 25.97% to 0.8%). Cellulose content increased from 52.45% to 67.31%, respectively [76] (Table 8).

In another study, three types of MW-assisted pretreatments were applied to *Miscanthus sinensis*: alkaline, acidic, and combined alkaline/acidic [77]. Several temperatures (in the range of 60 °C to 160 °C), and various periods of MW incidence (from 5 to 60 min) were examined. In the end, the cellulose, hemicellulose, and lignin contents were determined with and without the application of MW radiation (Table 8). With the application of microwaves, the amount of cellulose increased and that of lignin decreased in all pretreatments. Regarding the hemicellulose content, it decreased in acid pretreatment (from 31.3 to 20.7%) and in acid followed by alkaline pretreatments (from 31.3 to 18.4%) but increased slightly in alkaline pretreatment (from 31.3 to 32.9%). Hence, it was verified that the greatest removal of hemicellulose (from 31.3 to 18.4%) was in the microwave-assisted pretreatment, where the acid stage was followed by an alkaline one.

In another study, three types of pretreatments were applied to sugarcane bagasse: ultrasound, ammonia, and ammonia combined ultrasound [78].The results allowed us to conclude that the removal of hemicellulose was more effective in the pretreatment combined ammonia ultrasound (from 32.0 to 19.6%) (Table 8).

Recently, a study was carried out in which US treatment was applied to the hog plum (*Spondias mombin* L.). Three types of pretreatments were compared: ultrasound, nitric acid alone, and nitric acid combined with ultrasound [79]. The synergistic effect of the acid together with ultrasound significantly decreased hemicellulose and lignin contents—from 11.35 to 3.19% and from 35.28% to 10.18%, respectively—and led to an increase in cellulose content from 53.74 to 63.15% (Table 8).

**Table 8:** Effect of microwave radiation and ultrasound on lignin (L), hemicellulose (H), and cellulose (C) contents.

Biomass	Pretreatment	Operating Conditions	Initial Composition (%)	Composition after Treatment (%)	References
Sugarcane bagasse	Acid (H_2_SO_4_) + MW	P = 900 W f = 2.45 GHz ∆t = 5 min T = 190 °C	C: 52.45 H: 25.97 L: 12.72	C: 67.31 H: 0.8 L: 15.67	[76]
Winter wheat *(Miscanthus sinensis)*	Alkaline (NH_4_OH) + MW	P = 300 W ∆t = 15 min T = 120 °C	C: 42.7 H: 31.3 L: 17.4	C: 53.1 H: 32.9 L: 12.2	[77]
	Acid (H_2_SO_4_) + MW	P = 300 W ∆t = 30 min T = 140 °C	C: 42.7 H: 31.3 L: 17.4	C: 61.5 H: 20.7 L: 15.8	
	Acid + Alkaline (H_2_SO_4_ + NH_4_OH) + MW	∆t = 15 min (120 °C) +∆t = 30 min (140 °C)	C: 42.7 H: 31.3 L: 17.4	C: 69.7 H: 18.4 L: 10.4	
Sugarcane bagasse	US	P = 400 W f = 24 kHz ∆t = 45min, T = 50 °C	C: 38.0 H: 32.0 L: 27.0	C: 46.9 H: 29.3 L: 20.7	[78]
	Ammonia (10% *v*/*v*)	P = 400 W f = 24 kHz ∆t = 30min, T = 80 °C	C: 38.0 H: 32.0 L: 27.0	C: 50.4 H: 26.8 L: 19.8	
	Ammonia + US (10% *v*/*v*)	P = 400 W f = 24 kHz ∆t = 45min, T = 80 °C	C: 38.0 H: 32.0 L: 27.0	C: 56.1 H: 19.6 L: 18.2	
Hog plum (*Spondias mombin* L.)	US	P= 400 W f = 40 kHz ∆t = 60 min, T = 80 °C	C: 53.74 H: 11.35 L: 35.28	C: 60.19 H: 6.27 L: 13.17	[79]
	Nitric acid	P = 400 W f = 40 kHz ∆t = 60 min, T = 80 °C	C: 53.74 H: 11.35 L: 35.28	C: 55.27 H: 9.73 L: 24.14	
	Nitric acid + US	P = 400 W f = 40 kHz ∆t = 60 min, T = 80 °C	C: 53.74 H: 11.35 L: 35.28	C: 63.15 H: 3.19 L: 10.18	

The application of a pretreatment assisted by microwave or ultrasound commonly decreases the lignin and hemicellulose content and increases the cellulose content [60,68]. This is because hemicellulose, due to its amorphous character and vulnerable chemical structure, is more easily degraded and removed from biomass, while cellulose, due to its amorphous-crystalline structure and specific supramolecular organization, is more difficult to degrade and remove [60]. Accordingly, MW- and US-assisted pretreatments can be used for the targeted removal of hemicelluloses. Once the hemicellulose is removed from the biomass, the cellulose content increases.

#### 3.2.3. Effect on Cellulose Crystallinity Index

One of the objectives of any vegetable biomass pretreatment is the deconstruction of lignocellulosic matrix to decrease the degree of polymerization and structural organization of its macromolecular components. For cellulose, this is expressed as a decrease in its crystallinity. However, there are studies on pretreatments, assisted by microwaves and ultrasound, for which it was demonstrated that the crystallinity of cellulose increases. Several examples are set out below.

In the study on sugarcane bagasse, microwaves were applied in the pretreatments under acid or alkaline media and combining alkaline and acid chemicals. In all sugarcane bagasse pretreatments, the crystallinity index of cellulose increased from 53.4 to 58.79%, 65.29%, 53.44%, and 65.55% for the acid, alkaline, and alkaline followed by acid pretreatments, respectively (Table 9) [80]. In another study, in which only MW-assisted alkaline pretreatment was applied to water hyacinth, it was found that the cellulose crystallinity index decreased from 16 to 13% [81] (Table 9). Therefore, the effect that these pretreatment techniques have on the crystallinity index is not simple to predict.

A study in which a microwave expansion pretreatment (MEP) was used to extract hemicellulose from hemp stalk [74] consisted of two steps: MEP and microwave-assisted alkaline extraction (MAAE). The results showed that the crystallinity indexes of cellulose in the stem of the peeled stalk and in the stem with bark decreased from 44.96 to 42.83% and from 54.13 to 50.49%, respectively, after the MEP step (Table 9 only has the values related to the peeled hemp stalk with and without MEP).

A very promising route was developed on black tea waste to obtain microcrystalline cellulose by applying MW pretreatments [82]. Researchers have developed a microwave-assisted alkaline peroxide bleaching protocol consisting of three main steps, including (i) washing and drying of black tea residues; (ii) soda delignification for 2 min (5% NaOH) in the microwave reactor; and (iii) bleaching with alkaline peroxide performed in the microwave reactor for 30 s only in two cycles. The crystallinity index of cellulose increased from 56.68% to 76.86% in the control bleaching (two cycles) with alkaline peroxide at 55 ºC over 90 min without MW and to 88.77% with microwave-assisted alkaline peroxide bleaching for 0.5 min (two cycles) (Table 9). This work stated that the proposed route of microcrystalline cellulose production can be implemented on an industrial scale [82].

**Table 9:** Effect of microwave radiation on the cellulose crystallinity index in vegetable biomass.

Biomass	Pretreatment	Operating Conditions	Crystallinity Index After (%)	References
Sugarcane bagasse	No treatment		53.44	[80]
Sugarcane bagasse	Acid (H_2_SO_4_) + MW	P = 450 W f = 2450 MHZ ∆t = 5 min	58.79	[80]
	Alkaline (NaOH) + MW	P = 450 W f = 2450 MHZ ∆t = 5 min	65.29	
	Alkaline (NaOH) + Acid (H_2_SO_4_) + MW	P = 450 W f = 2450 MHZ ∆t = 10 min	65.55	
Water hyacinth	No treatment		16.0	[81]
	Alkaline (NaOH) + MW	P = N.d. ^3^ ∆t = 10 min T = 190 °C	13.0	
Hemp stalk	No treatment		44.96	[74]
	With MEP ^1^	P = 1100 W ∆t = 3 min T = 90 °C	42.83	
Black tea residues (*Camellia sinensis*)	No treatment		56.86	[82]
	Alkaline bleaching with peroxide	∆t = 90 min T = 55 °C	76.86	
	Alkaline bleaching with peroxide +MW ^2^	P = 1000 W ∆t = 0.5 min	88.77	

^1^ MEP—microwave expansion pretreatment. ^2^ Microwave-assisted delignification and bleaching. ^3^ N.d- Not determined.

In another study, in which the biomass was water hyacinth, the following ultrasonic pretreatments were proposed: (i) only with ionic liquid and (ii) with ionic liquid, combined with surfactant SDS (sodium dodecyl sulfate) [83]. The ionic liquid pretreatment also included a control assay without ultrasound. It was found that in all pretreatments, the crystallinity index of cellulose was increased (from 19.50 to 32.44%, 30.74%, and 28.50% only with ionic liquid, with ionic liquid assisted by ultrasound, and with ionic liquid with SDS and assisted by ultrasound, respectively) (Table 10).

In another work, enzymatic hydrolysis was applied just after ultrasonic pretreatment with kenaf powder [84]. Two processing routes were evaluated: (i) one with ionic liquid only and (ii) another with ultrasound-assisted ionic liquid. It was found that the crystallinity index decreased from 49.4 to 38.8% and to 31.5%, respectively (Table 10).

In another study, samples of eucalyptus wood were ground and dissolved in different media, namely soda solution, distilled water, and acetic acid solution, and then subjected to ultrasonic pretreatment for half an hour [85]. It was found that the crystallinity index of cellulose increased from 31.8 to 34.7%, 32.6%, and 33.4% in soda, water, and acetic acid, respectively (Table 10). It was also found that the incidence of ultrasound in wood resulted in a decrease in the content of alkali metals (potassium, calcium, and magnesium) in the processed material.

Recently, three ultrasonic routes were studied using residues of cupuaçu husk in aqueous, acidic, alkaline, and ionic liquid pretreatments [86]. It was found that with 30 min of ultrasound treatment, the crystallinity index increased from 54.3 to 60.0%, 63.3%, 57.0%, and 58.2% while applied in aqueous, acidic and alkaline solutions and in ionic liquids, respectively (Table 10). It was suggested that ultrasound increased crystallinity of all pretreated samples because of the removal of cellulose’s amorphous fraction.

**Table 10:** Effect of ultrasound on the crystallinity index of cellulose.

Biomass	Pretreatment	Operating Conditions	Crystallinity Index (%)	References
Kenaf powder	No treatment		49.4	[84]
	Ionic liquid		38.8	
	Ionic liquid +US	P = 35 W f = 24 kHz ∆t = 15 min T = 25 °C	31.5	
Water hyacinth	No treatment		19.50	[83]
	Ionic liquid		32.44	
	Ionic liquid + US	P = 100 W f = 20 kHz ∆t = 45 min T = 120 °C	30.74	
	Ionic liquid +US + SDS ^1^	P = 100 W f = 20 kHz ∆t = 45 min T = 120 °C	28.73	
Eucalyptus powder (*Eucalyptus grandis*)	No treatment		31.8	[85]
	Soda solution + US	P = 300 W f = 28 kHz ∆t = 30 min T = 50 °C	34.7	
	Water + US	P = 300 W f = 28kHz ∆t = 30 min T = 50 °C	32.6	
	Acetic acid + US	P = 300 W f = 28kHz ∆t = 30 min T = 50 °C	33.4	
Cupuaçu husk (*Theobroma grandiflorum*)	No treatment		54.3	[86]
	Water + US	P = 100 W f = 40 kHz ∆t = 30 min T = 35 °C	60.0	
	Acid (HCl) + US	P = 100 W f = 40 kHz ∆t = 30 min T = 35° C	63.3	
	Alkaline (NaOH) + US	P = 100 W f = 40 kHz ∆t = 30 min T = 35 °C	57.0	
	Ionic liquid +US	P = 100 W f = 40 kHz ∆t = 30 min T = 35 °C	58.2	

^1^ SDS—sodium dodecyl sulfate.

There are some US pretreatments reported in which the crystallinity index increases [80,83,85,86] and others in which it decreases [74,81,84]. Upon pretreatment, it is expected that the hydrogen bonds between the macromolecules will break, that the cellulose will be more exposed and more susceptible to treatment, and consequently that its crystallinity index will decrease [60,68]. However, there are multiple examples in which the crystallinity index increased. Several hypotheses have been raised to explain the characteristics of cellulose crystallinity in MW and US treatments, which include the most common opinion that the action of microwaves on the amorphous zone of cellulose is predominant in relation to the crystalline zone [80]. Partial amorphization of crystalline cellulose can explain the decrease in cellulose crystallinity. On the other side, it is reported that the crystallinity index increased because ultrasound removed lignin and hemicellulose and the cellulose became more exposed to reactions affecting the amorphous counterpart [85]. In a recent review on ultrasonic processing applied to food waste for value-added products, in most of the examples presented, the crystallinity index increased, and the idea that this was because its relative content increased due to the partial elimination of the amorphous counterpart was defended [69].

#### 3.2.4. Effect on the Solubilization of Organic Matter

In order to improve the anaerobic digestion of microalgae biomass, researchers compared various types of physical pretreatments, including the incidence of microwaves (P = 900 W, ∆t = 3 min) and ultrasound (P = 70 W, ∆t = 30 min) [87]. These researchers concluded that soluble organic matter, soluble proteins, soluble carbohydrates, and soluble lipids increased with microwave-irradiation-assisted pretreatment 8-, 18-, 12-, and 2-fold, respectively. With ultrasonic pretreatment, soluble organic matter, soluble proteins, soluble carbohydrates, and soluble lipids increased 7, 12, 9, and 3-fold, respectively (Table 11). This work had as its purpose the production of methane. After anaerobic digestion, the pretreatment with microwave incidence resulted in an increase in methane yield of 21%. However, with ultrasound pretreatment, no significant increase in methane yield was registered.

The effect of ultrasound on anaerobic digestion in order to produce methane in two types of microalgae was examined [88]. The best increase in soluble chemical oxygen demand (sCOD) for the microalgae *Tetraselmis suecica* was achieved with only 5 s of sonication time and reached 5.13%. For the microalgae *Nannochloropsis oceanic*, the best sCOD was 18%, achieved with 54 s (Table 12). It is concluded that the best algae for methane production is *Tetraselmis suecica*.

By analyzing the data in Table 11 and Table 12, it can be concluded that microwaves and ultrasound contribute to the breakdown of the cell walls of lignocellulosic materials and consequently to the transfer of organic matter to the soluble phase [40,43,50], which is crucial in the production of biofuels, such as methane. Recently, a review on ultrasonic processing of food waste, with 26 studies on the influence of ultrasound on chemical oxygen demand (sCOD), was reported [69].

#### 3.2.5. Effects on Hydrolysis and Reduction of Sugar Production

The main obstacle to the production of biofuels from lignocellulosic biomass is difficulty in hydrolyzing their structural polysaccharides into simple sugars, a step that is necessary for further effective fermentation. In the work of Khamtib et al. [89], an acid pretreatment combined with microwave radiation was used on oil palm trunk in order to produce hydrogen. In this work, the optimal conditions for the microwave-assisted pretreatment were designed using the response surface methodology (RSM). With only 7.5 min of microwave incidence (P = 450 W), a high yield was achieved in various sugars—glucose, xylose, and arabinose—with a glucose yield of 8.95 g/L (Table 13).

In the work of Zhu et al. [90], the *Miscanthus* grass was examined in several microwave-assisted pretreatments at various temperatures and in different media, including water and aqueous, acidic, and alkaline solutions. It was found that for all pretreatments, regardless of the medium used, the sugars increased with the increase in temperature up to 180 °C but then decreased with further increase in temperature. Another conclusion was that the maximum sugar yield (73%) was achieved at 180 °C, with acid pretreatment (H_2_SO_4_) assisted by microwaves. Comparing this result, obtained with the incidence of microwaves (incidence time 20 min, but does not refer to power) to that of pretreatment with conventional heating, the researchers stated a 17-times increase in yield achieved in half the time. In another study, the alkaline pretreatment combined with ultrasound was applied to sugarcane bagasse before the hydrolysis process for the production of bioethanol [91]. It was found that ultrasonic alkaline pretreatment of only 5 min (P = 35 kHz and temperature of 65 °C) increased the production of sugars from 3.62 g/L to 5.78 g/L.

In another study, the residues of cupuaçu (*Theobroma grandiflorum*) bark were used as raw biomass for glucose production with three types of ultrasonic pretreatment in water and aqueous, acidic, and alkaline solutions [86]. The glucose productions with the different pretreatments were 8.44 g/L, 9.90 g/L, and 6.08 g/L for the ultrasonic pretreatments in water, in acidic, and alkaline solutions, respectively, and only 3.14 g/L without ultrasonic pretreatment (Table 13). Thus, it was concluded that the best glucose yield was achieved in the acid pretreatment. It should be noted that the sugars obtained were subsequently used to prepare 5-hydroxymethylfurfural (5-HMF) and furfural (FF), and the results are discussed in Part III of this review.

Another group of researchers optimized, using central composite methodology, the alkaline microwave-assisted pretreatment of rice straw [1]. After the sugars were obtained, they were converted into 5-hydroxymethylfurfural (5-HMF). The optimal conditions found for saccharification were ultrasound power 681 W, temperature 120 °C, sodium hydroxide concentration 0.54 M, and pretreatment time only 3 min (Table 13). Under these conditions, they were able to obtain a maximum yield of 350 mg of sugars per gram of treated rice straw and a glucose yield of 255 mg per gram of treated rice straw. After obtaining the sugars, enzymatic hydrolysis was performed with a microwave for 30 min at 120 °C using a compound of titanium magnetic silica nanoparticles as a catalyst. Under these conditions, the researchers were able to produce 5-HMF with 41.1% yield in half an hour.

An innovative route was hydrotropic pretreatment assisted by microwave radiation, which was applied to pine chips, beech chips, and wheat straw [92]. It should be noted that this hydrotropic pretreatment, assisted by microwaves, was carried out for one hour and at a pressure of 117 Psi and that the hydrotrope used was sodium cumene sulfonate (NaCs). One of the objectives of this study was to investigate the influence of this hydrotropic pretreatment on the enzymatic hydrolysis of cellulose. Table 13 shows the results obtained after 72 h of enzymatic hydrolysis, without pretreatment, and with pretreatment in water—assisted by microwave and with microwave-assisted pretreatment with NaCs—for the three types of biomass. The main conclusions were formulated as follows: (i) pine chips are more vulnerable to hydrolysis because they exhibited the highest yield (77 mg of glucose/g of biomass); (ii) wheat straw was totally resistant because it had zero yield; and (iii) beech chips and wheat straw with hydrotropic microwave-assisted pretreatment reached yields higher than 500 mg of glucose/g of biomass, 515.5 mg glucose/g biomass, and 557.3 mg glucose/g biomass, respectively (Table 13).

Combining several types of pretreatments is also a good strategy for deconstructing biomass. Recent work reports the combined chemical pretreatment (using a deep eutectic solvent), physical pretreatment (using microwave incidence), and enzymatic pretreatment in a single stage to produce bioethanol from rice straw [93]. In this work, after optimization using RSM, the efficiency of sugar production increased by 1.67 times with the application of MW pretreatment.

**Table 13:** Effect of ultrasound and microwave pretreatments on the released glucose content.

Biomass	Pretreatment	Operating Conditions	Glucose Production	Reference
Oil palm trunk	Acid (H_2_SO_4_) +MW	P = 450 W ∆t = 7.5 min	8.95 mg/L	[89]
Rice straw	Alkaline (NaOH) + MW	P = 681 W ∆t = 3 min	255 g/g ^1^	[1]
Pine chips	No treatment		77.3 mg/g ^1^	[92]
	water + MW	P = 600 W ∆t = 60 min Pressure = 117 Psi	81.5 mg/g ^1^	
	NaCs^2^ + MW	P = 600 W ∆t = 60 min Pressure = 117 Psi	107.8 mg/g ^1^	
Beech chips	No treatment		35.0 mg/g ^1^	[92]
	water + MW	P = 600 W ∆t = 60 min Pressure = 117 Psi	278.0 mg/g ^1^	
	NaCs ^2^ + MW	P = 600 W ∆t = 60 min Pressure = 117 Psi	515.5 mg/g ^1^	
Wheat straw	No treatment		0.0 mg/g ^1^	[92]
	water + MW	P = 600 W ∆t = 60 min Pressure = 117 Psi	435.8 mg/g ^1^	
	NaCs ^2^ + MW	P = 600 W ∆t = 60 min Pressure = 117 Psi	557.3 mg/g ^1^	
	No treatment		3.14 g/L	[86]
Residues of cupuaçu (*Theobroma* *grandiflorum*)	water + US	P = 100 W f = 24 kHz ∆t = 30 min	8.44 g/L	
	Acid (HCl) + US	P = 100 W f = 24 kHz ∆t = 30 min	9.90 g/L	
	Alkaline (NaOH) + US	P = 100 W f = 24 kHz ∆t = 30 min	6.08 g/L	

^1^ mg/g of treated biomass. ^2^ Hydrotropic pretreatment with sodium cumene sulfanate (NaCs) at a pressure of 117 Psi.

It can be concluded, as follows from the examples given, that a pretreatment assisted by microwaves or by ultrasound can have a decisive role in obtaining sugars for subsequent hydrolysis (a theme that will be discussed in Part III of this review) with a view to obtaining biofuels or bio-based products.

## 4. Microwaves and Ultrasound on the Route of Value-Added Products (Part III)

Currently, there are many publications on the application of microwave radiation and ultrasound as a technique for the pretreatment of lignocellulosic biomass, especially directed at the production of biofuels. However, in the current context of bioeconomy and biorefinery, these techniques are gaining more and more importance, and conventional operations—with a view to the preparation of biochemicals and value-added products—are progressively engaging these two techniques (separately or combined). Thus, two conventional operations—such as liquefaction and solvent extraction, which aim to transform waste biomass into value-added products—can be substantially promoted while applying microwave radiation and ultrasound.

### 4.1. Liquefactions

The thermochemical conversion of lignocellulosic biomass can be carried out by gasification, pyrolysis, or liquefaction. Gasification is carried out at very high temperatures (up to 1000 °C), and as a product, a gaseous mixture composed mainly of CO and H_2_ is obtained [94]. Pyrolysis, similarly to gasification, is also a method of thermal decomposition, but the main difference is that pyrolysis is carried out in an oxygen-free environment. As for the liquefaction of lignocellulosic biomass, there are two types: the one performed at high pressures (5–10 MPa) and another performed at low pressures and moderate temperatures (100–250 *°*C) [94]. This liquefaction under conditions of moderate temperature and pressure has attracted much research and is called solvolysis.

Conventional liquefactions (or solvolysis) are carried out in a reactor in which the heating is conducted using an oil bath or in another way at moderate pressures, using polyhydric alcohols as solvents and catalysts (acid or basic). The interest of liquefactions is, as the terminology suggests, to transform lignocellulosic biomass into liquid mixtures for further preparation of value-added products—for example, polyurethane foams or phenolic resins.

Below are some examples of how ultrasound and microwaves are promising techniques for carrying out liquefactions.

#### 4.1.1. Ultrasound-Assisted Liquefactions

A group of researchers studied the liquefaction of various types of municipal wood waste, including medium-density fiber (MDF) boards and veneered particleboard, using ultrasound [95]. In addition to these residues, wheat straw was also liquefied. The time for conventional liquefaction of MDF was 90 min; with ultrasound application, it took only 10 min. Therefore, with the introduction of ultrasound, reaction times were reduced 9 times (Table 14). Another important conclusion was that the use of ultrasound had no influence on the number of hydroxyl groups of the polyol obtained, so the polyol remained suitable to produce polyurethane foams. In this work, an ultrasound probe of P = 400 W and f = 24 kHz was used, the solvent used in liquefaction was a mixture of diethylene glycol and glycerol, and the catalyst was sulfuric acid [95].

Ultrasound was also used in the liquefaction of cork powder to produce polyurethane foams [96]. In this work, the researchers considered the kinetics of the liquefaction reaction as being of the first order and concluded that liquefaction with ultrasound increased the speed of the reaction by up to about 4.5 times in relation to the liquefaction performed with the conventional method. They also verified that without ultrasound, 135 min is needed to achieve the best yield of 95%, and that with the application of ultrasound, the liquefaction time was only 75 min, with this time achieving a yield higher than 98% (Table 14). Another important conclusion of this work was that the increase in the amplitude of the ultrasound consequently decreased the number of hydroxyl groups of the polyol, but polyols continue to be suitable for producing polyurethane foams. In this work, an ultrasound probe of P = 400W and f = 24 kHz was used. The solvent was a mixture of ethylene glycol and 2-ethylhexanol, and the catalyst was *p*-toluene sulfonic acid (PTSA).

**Table 14:** Examples of ultrasound- and/or microwave-assisted liquefactions compared to the respective conventional liquefactions.

Biomass	Type of Liquefaction	Liquefaction time (min)	Liquefaction Yield (%)	Reference
Medium density fiberboard (MDF)	Conventional	90	93.8	[95]
	US	10	94.9	
Wheat straw	Conventional	90	94.4	
	US	15	95.4	
Veneered particleboard	Conventional	120	95.0	
	US	20	96.0	
Cork powder	Conventional	135	95.0	[96]
	US	75	98.0	
Poplar sawdust	Conventional	….	….	[97]
	MW	7	100	
Corn stover and corncob	Conventional	….	….	[98]
	MW	20	95	
Bamboo wastes	Conventional	….	….	[99]
	MW	7	96.7	
Bamboo sawdust	Conventional	….	….	[100]
	MW	8	78	
Fir sawdust	Conventional	60	….	[101]
	MW + US	20	91	
	Conventional	60	….	[102]
	MW + US	<20	….	

#### 4.1.2. Microwave-Assisted Liquefactions

One example of liquefaction assisted by microwaves is the liquefaction of poplar sawdust in only 7 min (Table 14) [97]. In this work, microwave heating was conducted for 2 min at 500 W and then followed by another 5 min of microwave incidence at a power of 300 W. The catalyst was PTSA, and a mixture of glycerol with glycols was used as a solvent.

To study the influence of biomass type on its liquefaction results assisted by microwaves, the liquefactions of five different residual biomass were compared: corn stover, rice straw, wheat straw, cotton stalk, and corncob [98]. The solvent used for these liquefactions was ethylene glycol, and the catalyst was sulfuric acid. The results showed that all biomass was liquefied, with a good yield of 71 to 82%, in the first five minutes of liquefaction; corn stover and corncob were liquefied to 95% in only 20 min of liquefaction [98] (Table 14).

In the microwave-assisted liquefaction of bamboo, five solvents were evaluated, namely glycerol, polyethylene glycol, methanol, alcohol, and water [99]. Using sulfuric acid as a catalyst, it was possible to achieve a yield of 96.7% with glycerol in 7 min of liquefaction assisted by a microwave power of 550 W (Table 14).

Recently, microwave-assisted liquefaction of bamboo sawdust was carried out in order to use the polyol obtained in the production of polyurethane foams [100]. In this work, the optimal conditions were achieved with a time and a liquefaction yield of 8 min and 78%, respectively, using sulfuric acid as the catalyst and diethylene glycol as the solvent. (Table 14). All the reagents were placed in a bath with an ultrasound with a power 253 W for 15 min and then put into the microwave oven for 8 min. It should be noted that the polyols obtained in liquefaction have been successfully used in the preparation of polyurethane foams possessing flame resistance.

#### 4.1.3. Liquefactions Assisted Simultaneously by Microwaves and Ultrasound

In 2016, a liquefaction assisted by microwaves and ultrasound was carried out for the first time [101]. In this liquefaction assay, spruce sawdust was processed in a mixture of polyethylene glycol (PEG 400) and glycerol, using sulfuric acid as the catalyst. The liquefaction yield reached 91% under optimal conditions. Compared to conventional liquefaction, this trial allowed the reduction of solvent consumption by half and reduced the liquefaction time from 60 to 20 min (Table 14). The parameters related to microwaves and ultrasound were adjusted as follows: first, heating with microwaves at 250 W, for 2 min; then, the microwave was set to 60 W for 18 min. Ultrasound treatment followed the entire liquefaction process induced by MW irradiation.

In another liquefaction of spruce sawdust assisted simultaneously by microwaves and ultrasound, n-butanol and sulfuric acid were used as solvent and catalyst, respectively [102]. With this strategy, the researchers claimed to achieve liquefaction within less than 20 min and an increase in the percentage of liquefaction of 5.24% compared to conventional liquefaction (Table 14).

Several studies point out the main disadvantages of conventional liquefaction compared to microwave-assisted (or/and ultrasound) liquefaction. There are three fundamental disadvantages: longer reaction time, lower rate of liquefaction, and the use of large amounts of solvents, which is environmentally inadvisable [103]. It should also be noted that in conventional liquefactions, the heating system (usually an oil bath or other heating device) heats up very slowly, which results in high energy consumption.

A recent review describes some microwave-assisted liquefaction works involving agricultural and forestry residues with a view to producing value-added products, namely polyurethane foams and phenolic resins [103]. These researchers concluded that microwave liquefaction of lignocellulosic biomass at the laboratory bench scale is an efficient and ecologically sound route and suggest moving towards pilot-scale studies. However, they suggested that more studies should to be conducted in the future on the ability of biomass to absorb microwave radiation [103]. In this sense, it is of crucial importance to carry out a study of the dielectric constant of biomass.

### 4.2. Microwave-Assisted and/or Ultrasound-Assisted Extractions

Extractions are unit operations that aim to separate the bioactive compounds from the solid matrix. Conventional extraction, usually performed via Soxhlet extraction, has several disadvantages, such as high time of operation, use of large amounts of solvents, and possibility of degradation of the compounds [19,104,105,106]. Faced with these disadvantages, it became necessary to find an ecological and effective method of extraction, and therefore, the techniques of solvent ultrasound-assisted and/or microwave-assisted extraction are emerging. As was pointed out in Part I of this review, ultrasound (by means of cavitation) and microwaves (by means of dipole rotation and ion conduction) lead to the rupture of cell walls, thereby increasing the mass transfer between the solid and the liquid phases. For these reasons, extractions that use these microwaves and ultrasound techniques prove to be quite effective. It should be noted that ultrasonic extractions have been used successfully in the food industry for many years [15].

Given that there are many publications on microwave and/or ultrasound extraction, it was decided to limit the examples that aim to obtain phenolic compounds from lignocellulosic biomass. The referred phenolic compounds include phenolic acids, flavonoids, and tannins [69]. These compounds extracted from lignocellulosic biomass are currently valued in several areas, mainly in the food and pharmaceutical industries [106]. Examples of ultrasonic and microwave extraction are discussed below as autonomous or combined techniques.

In 2015, microwave extraction of phenolic compounds from *Eucalyptus robusta* was reported [104]. In this work, authors pointed out that the main advantages of microwave extraction, compared to conventional extraction, are reduction in extraction time and, consequently, lower energy consumption and lower probability of degradation of the extractive compounds. However, they state that the difficulty of applying this technique is due to the lack of knowledge of the optimal conditions (the irradiation time, the power of the microwave apparatus, and the sample/solvent ratio). The lack of knowledge of the parameters makes it difficult to optimize extraction, as these parameters are scarce in the literature and because the same parameters can vary depending on the biomass in question. In this study, such phenolic compounds as flavonoids and proanthocyanidins were extracted from *Eucalyptus robusta* [104]. For this extraction with water, only 3 min of microwave incidence time at 600 W was required (Table 15). The ideal extraction conditions (irradiation time, microwave power, and sample/solvent ratio) were found using the response surface methodology (RSM). In conclusion, it was demonstrated that the factor with the greatest effect on the extraction yield is the sample/solvent ratio and that the one that has the least influence is the irradiation time. This microwave-assisted extraction of phenolic compounds from *Eucalyptus robusta* was compared to ultrasound-assisted extraction a few years later, in 2017 [104,105]. In that work, the researchers pointed out as the main advantage of ultrasonic extraction the fact that it is a fast, simple, and effective technique in which the decomposition of the compounds is minimized while operating at 250 W of MW power and for 90 min of incidence time (Table 15). It should also be noted that in this work, several solvents were evaluated, such as water, ethanol, acetonitrile, and ethyl acetate. However, water proved to be the best solvent. According to results obtained on the amounts of polyphenolics and their antioxidant activity, this developed extraction route was claimed as a green technique for the extraction of phenolics from that eucalyptus species. In this work, the researchers also used the RSM as a computational tool to find the optimal operating conditions. Microwave-assisted extraction (MAE) and ultrasound-assisted extraction (UAE) of polyphenolics in aqueous ethanol solutions was also reviewed as more effective tools than pressurized liquid extraction (PLE), supercritical fluid extraction (SFE) [106].

Another work on the extraction of active compounds from eucalyptus species (*Eucalyptus globulus*) was carried out by Gullón et al. [107], who performed five types of extractions to compare: enzyme-assisted, microwave-assisted, ultrasound-assisted, eutectic liquid, and conventional extraction. Table 15 shows only microwave-assisted, ultrasound-assisted, and conventional extraction results. In this work, the extraction time in conventional extraction was 225 min, which was reduced to 90 min and to 7 min using ultrasound and microwaves, respectively. Given these extraction times, the researchers concluded that the energy consumption of ultrasound-assisted extraction was half that of conventional extraction and that the energy consumption of microwave-assisted extraction was thirteen times lower than that of conventional extraction. It should be noted that 26 phenolic compounds were identified in these extracts, and these researchers considered microwave extraction a promising green extraction method [107].

To compare the two techniques, microwave-assisted extraction and ultrasound-assisted extraction, they were applied to lemon peel residues [108]. After optimization employing RSM, the ultrasound-assisted extraction (amplitude 38%) took 4 min and the microwave-assisted extraction (P = 140 W) took only 45 s to reach similar extraction results (Table 15).

Conventional extraction of phenolic compounds allowed the identification of 36 flavonoids from *Spatholobus suberectus* (an herb used for various medicinal purposes) [109]. In this work, the laboratory extraction with a Soxhlet apparatus took six hours, whereas with ultrasound and microwaves, the extraction took one hour and half an hour, respectively. However, under simultaneous use of microwave and ultrasound, it was found that similar extraction results can be achieved for 7.5 min only, thus demonstrating the synergetic effect of MW and US (Table 15).

Recently, a study on the extraction of phenolic compounds from the leaves of coriander (*Coriandrum sativum* L.) was published employing MW [22]. In this work, several solvents were evaluated, such as ethanol, acetone, and water. Ethanol (at 50%) proved to be the most suitable solvent for polyphenolics extraction. In this work, microwave and conventional extraction were compared, and the operating parameters were optimized by RSM. The optimized parameters were related to the ethanol concentration, irradiation time, microwave power, and liquid/solid ratio. Regarding the irradiation time and microwave power, values from one to five minutes and from 100 to 900W were tested. It was concluded that the maximum extraction occurred in four minutes at 500 W (Table 15).

**Table 15:** Comparison between various types of solvent extraction to obtain phenolic compounds from different biomasses.

Biomass	Type of Extraction	Power	Solvent	Extraction Time (min)	Reference
*Eucalyptus robusta* *	MW	600 W	Water	3	[104]
*Eucalyptus robusta* *	US	250 W	Water	90	[105]
*Eucalyptus globulus*	Conventional	Medium	Ethanol 56% (V:V)	225	[107]
	US			90	
	MW			7	
Lemon peel residues *	US	Amplitude 38%	Ethanol:Water55:45	4	[108]
	MW	140 W		0.75	
*Spatholobus suberectus*	Conventional		100% Methanol	360	[109]
	US	30–250 W	70% Methanol	60	
	MS	100–500 W	70% Methanol	30	
	MS + US	100–500 W 30–250 W	Methanol 30–100% + Pure ethanol	7.5	
(*Coriandrum sativum* L.) *	MW	500 W	50% Ethanol	4	[22]

* RSM—response surface methodology.

### 4.3. Factors Influencing Microwave- and Ultrasound-Assisted Extraction

It can be concluded through the examples discussed that the main factors that influence microwave-assisted extraction are irradiation time, the power of the microwaves, the frequency of the microwaves, and temperature [110]. It should be taken into account that when the irradiation time is increased, it can consequently increase the extraction yield, but this can lead to unwanted degradation of the analytes [110]. As for the irradiation time and the power of the microwaves, these factors determine the consumption of energy, and it is advisable to keep microwave power low or medium [110]. The factors that influence ultrasound-assisted extraction include sonication time, ultrasound power, ultrasound frequency, and temperature [110].

### 4.4. Emerging Routes in Extraction

In a recent review, four emerging techniques for the extraction of phenolic compounds were compared: (1) microwave-assisted extraction, (2) pressurized liquid extraction, (3) supercritical fluid extraction, and (4) ultrasound-assisted extraction. A set of different biomasses was employed for each of these extraction techniques [106]. The main conclusions were formulated as follows: (1) such extraction processes as assisted by microwaves, pressurized liquid, supercritical fluid, and ultrasound are effective techniques for obtaining phenolic compounds; (2) for microwave-assisted, pressurized liquids and ultrasonic extractions, the most appropriate solvent is ethanol–water; (3) in the case of supercritical-fluid-assisted extraction, the best co-solvent is ethanol; (4) microwave-assisted extraction and pressurized liquid extraction require higher temperatures than other methods (but care is recommended to avoid degradation of the compounds); (5) the most promising extraction technique is microwave extraction followed by ultrasound-assisted extraction and extraction by pressurized liquid and supercritical fluid (this sequential ordering was performed based on total flavonoids extraction) [106]. These researchers suggest—as examples to be applied in the future—two routes, namely: (1) extraction with ultrasound combined with extraction with pressurized liquid or else extraction with ultrasound combined with extraction by supercritical fluid; (2) supercritical fluid extraction followed by microwave extraction or ultrasonic extraction followed by extraction with pressurized liquid [106].

In the context of promising extraction routes, other researchers propose the development of the following combinations between these advanced techniques that can exhibit promising results: ultrasound-assisted enzymatic extraction, microwave-assisted enzymatic extraction, and ultrasonic microwave-assisted extraction [111]. These researchers, in addition to suggesting these combinations, also present success stories in which these combinations have already been tried.

## 5. Sonocatalysis of Lignocellulosic Biomass (Part IV)

The area of chemistry that studies the use of ultrasound in chemical reactions is called sonochemistry. From a historical point of view, the first time that ultrasound was used to increase the speed of a chemical reaction was in 1927, as reported by Richards and Loomis [62]. However, it was only later, in the 1970s, that sonochemistry was born as an emerging area of chemistry [27,58]. Sound is rarely associated with chemical reactions [27]. However, statements of the principles of sonochemistry can be found in the synthesis of chemical compounds: use less dangerous chemicals and more ecological solvents; increase the yield of chemical reactions; minimize energy consumption in chemical reactions; and use renewable raw materials [112]. There is a strong parallel between these 4 principles of sonochemistry and the 12 principles of green chemistry [112,113].

The interception of sonochemistry with catalysis results in sonocatalysis [64]. These two areas, sonochemistry and sonocatalysis, are emerging areas in the current context of bioeconomy and biorefinery. Sonocatalysis aims to find catalysts whose activity is triggered by ultrasound and which have access to complex compounds, which is relevant when the goal is to deconstruct lignocellulosic biomass, given its recalcitrant nature [67].

The main reactions that are catalyzed by ultrasound: hydrolysis, hydrogenation, and oxidation are presented and discussed [64,67]. These contribute to the deconstruction of lignocellulosic biomass in order to obtain biochemicals and biofuels.

### 5.1. Examples of Hydrolysis

An example of the application of hydrolysis in lignocellulosic biomass consisted of applying an ionic liquid and ultrasound to glucose, cellulose, and local bamboo to produce 5-hydroxymethylfurfural (5-HMF) [114]. In this work, furfural yields of 43%, 31% and 13%, respectively, were obtained from glucose, cellulose, and local bamboo, in less than 10 min. It should be noted that the conventional process (without ultrasound) takes 3 h (Table 16). The importance of reducing 5-HMF production time is due to the fact that this compound is on the list of the top ten bio-based products [1].

Another study deals with several ionic liquids that were tested to—using ultrasound—reduce sugars from two agricultural residues, corn straw and soybean straw [115] (Table 16). According to the results of this work, the basic conclusions were drawn as follows: (i) the best ionic liquid was 1-H-3-methylimidazolium chloride ([HMIM]Cl); (ii) the presence of ultrasound greatly improved the yield of obtained sugars; (iii) the protocol for obtaining the ionic liquid is simple and economical, and the ionic liquid, in addition to being a good solvent, also has good catalytic activity. This route of preparation of reducing sugars can be a promising step in the production of biofuels [115].

Another work of acid hydrolysis, assisted by ultrasound, was the synthesis of furfural (FF) from cellulose, under mild temperature conditions [116]. In this work, several acids were examined (nitric, sulfuric, hydrochloric, and oxalic) under different temperatures (from 30 to 70 °C) and different ultrasonic amplitudes (from 30 to 70%). It was possible to obtain a yield of 78% in the production of furfural with diluted nitric acid at 60 °C for 60 min with 50% ultrasound amplitude (Table 16). This route of furfural production from cellulose, with the addition of dilute nitric acid and ultrasound, can be applied industrially. It should be noted that furfural has a wide range of applications, including pharmaceuticals and polymers.

Another work involving acid hydrolysis was the production of reducing sugars, using microwaves and ultrasound, from various industrial residues of potato peel [117]. The industrial residues of potato peel used for the sugars production were “potato flour”, “wet potato sludge”, and “dry potato sludge” supplied by a company that generates about 20 tons per day. The sugar yields were 61% using microwaves and 70% and 84% with low- and high-frequency ultrasound, respectively (Table 16). Using reducing sugars obtained from industrial potato peel residues, biofuels are being produced.

A recent work of acid hydrolysis with ultrasound was the synthesis of 5-hydroxymethylfurfural (5-HMF) from banana peels [23] (Table 16). This work has two parts. In the first part, the delignification was performed with alkaline pretreatment, and in the second, acid hydrolysis was performed. Both experimental parts were assisted using ultrasound. In this investigation, several operation parameters were studied and optimized, and the results of both experimental parts, with and without the use of ultrasound, were compared. In conclusion, ultrasound reinforced both delignification and acid hydrolysis.

Another recent work was the preparation of 5-HMF and FF, using ultrasound, from the sugars previously obtained from cupuaçu husk [86]. This pretreatment was described previously (in Section 3.2.5). In this study, the best results were 12.94% and 48.84% in the synthesis of 5-HMF and FF, respectively, in one hour with ultrasound (T = 140 °C) using ionic liquid (Table 16).

In the three examples of hydrolysis presented [1,23,86], different routes for the production of 5-HMF have been suggested, but it is not obvious which is the best route, as it would require a more detailed analysis of factors other than yield, such as consumption power.

### 5.2. Examples of Hydrogenations

An example of ultrasound application to hydrogenation in lignocellulosic biomass was the work of hydrogenation of D-fructose with ultrasound to produce D-mannitol [118]. To achieve this goal, three catalysts were studied, namely Cu/SiO_2_; Raney-Ni, and CuO/ZnO/Al_2_O_3_ (Table 16). It was found that not all catalysts increased the hydrogenation rate, and it was concluded that the catalyst with the best performance in the production of D-mannitol in the presence of ultrasound was Cu/SiO_2_. It should be noted that D-mannitol is a sugar that exists in nature but whose extraction is not profitable, and this route of obtaining from D-fructose through ultrasound may be a more economical method of obtaining it.

Furthermore, lignin isolated from *Miscanthus giganteus* by acid hydrolysis and by basic hydrolysis was subjected to catalytic depolymerization via two reaction routes under selected thermal conditions and applying ultrasound [119]. In this work, the researchers also tested three different classes of nickel-containing catalysts (Table 16) and concluded that depolymerization was more efficient in lignin obtained via basic hydrolysis than by acid, and the catalysts exhibited lower catalytic performance under ultrasonic conditions than with conventional heating. This work shows that finding efficient and more ecological catalysts for the treatment of lignocellulosic biomass under ultrasonic conditions is a challenge.

Commonly, fatty acid methyl esters (FAMEs) are used to produce biodiesel through hydrogenation via conventional catalytic transfer under high temperatures and pressures. Recently, a group of researchers has developed an innovative route [120] using ultrasound to intensify the hydrogenation, which occurs at 35 °C for 120 min and has a yield of 78.66% in hydrogenated FAMEs. In this work, La-doped nickel boride amorphous alloy (Ni–La–B) was used as a catalyst, sodium borohydride was used as a hydrogen donor, and water was used as a solvent.

### 5.3. Examples of Oxidations

Regarding oxidations applied to lignocellulosic biomass in the field of sonochemistry, two case studies may be highlighted in which cellulose nanocrystals with high carboxylate content were prepared, one from cotton pulp and the other from hardwood kraft cellulose [121,122] (Table 16). In these two works, TEMPO was used as the catalyst (2,2,6,6-tetramethyl-piperidine-N-oxyl), which catalytically oxidizes cellulose with a high yield of carboxylated derivative [122].

Recently, Ayoub et al. [123] reported a new route for the production of maleic acid from FF using high-frequency ultrasound (525 to 565 kHz) which does not require the use of any catalyst. It should be noted that the search for the best route to produce maleic acid from furfural has motivated many studies, as this compound is a very important intermediate in the chemical industry. In this innovative route, a yield of 92% maleic acid was achieved under mild oxidizing conditions with hydrogen peroxide at a temperature of 42 °C without the use of a catalyst. This is a promising route, as conventional routes require complex catalysts and sometimes require high temperatures (Table 16). Several examples of ultrasound-assisted oxidation and hydrolysis reactions to produce nanocrystals from lignocellulosic biomass were recently reviewed [124].

**Table 16:** Some examples of sonocatalysis in hydrolysis and hydrogenations applied to lignocellulosic biomass.

Reaction	Biomass	Operating Conditions	Product	Main Conclusion	Reference
Hydrolysis	Bamboo *(Gigantochloa scortechinii)*	**Ultrasound** 20 kHz, 300 W 10 min, 140 °C **Catalyst:** ionic liquid CrCl_3_	5-HMF	From 3 h from the conventional route to 10 min.	[114]
	Soybean straw and corn straw	**Ultrasound** Bath, 120 min, 70 °C **Catalyst:** ionic liquid ([HMIM] Cl)	Reducing sugars	Simple and economical approach.	[115]
	Cellulose	**Ultrasound** 20 kHz, 60 min, 30 °C **Catalyst:** Diluted HNO_3_	FF	Simple synthesis, in 60 min, with yield 78%.	[116]
	Potato starch waste	**Ultrasound** 20 kHz and 500 kHz, 120 min, 60 °C **Catalyst:** H_2_SO_4_	Reducing sugars	70% yield with 20kHz ultrasound and 84% yield with 500kHz.	[117]
	Banana peels	**Ultrasound** 20 kHz, 240 watts **Catalyst:** H_2_SO_4_	5-HMF	Production of 50 g/L 5-HMF for 1 h	[23]
	Pretreated sugars obtained from cupuaçu husk (*Theobroma grandiflorum*)	**Ultrasound** It doesn’t mention power. 60 min, 140 °C **Catalyst:** ionic liquid $\;\left[BMIM\right]\left[Br\right]$	FF 5-HMF	Synthesis with yield of 12.94%, in 5-HMF and 48.84% in FF, in one hour	[86]
Hydrogenation	D- Fructose	**Ultrasound** 20 kHz, 50 W 20 min, 110 °C **Catalysts:** Cu/SiO_2_ Raney-Ni, CuO/ZnO/Al_2_O _3_	D-mannitol	Cu/SiO_2_ was the catalyst with best performance.	[118]
	Lignin from *Miscanthus giganteus*	**Ultrasound** 35 kHz, 6 h, 25 °C **Catalysts:** Fe_3_O_4_(NiAlO)_x_, Fe_3_O_4_(NiMgAlO)_x_, ionic liquid [BMIM]OAc	Low molecular weight compounds	The performances of the catalysts, under ultrasonic conditions, were inferior to those exhibited with conventional heating.	[119]
	Gross FAMEs	**Ultrasound** 40 kHz, 120 W, 35 °C **Catalyst:** Amorphous alloy of doped nickel boride with La Li-La-B.	hydrogenated FAMEs	Intensification of hydrogenation by catalytic transfer due to the incidence of ultrasound. The same catalyst can be used at least 5 times.	[120]
Oxidation	Cotton pulp	**Ultrasound** 40 kHz, 300 watts **Catalyst:** TEMPO (2,2,6,6-tetramethyl-piperidine-N-oxyl)	Nanocellulose with high COOH content	Cellulose nanocrystals stable in water	[122]
	Hardwood Kraft Pulp	**Ultrasound** 68 and 170 kHz, 1000 W **Catalyst:** TEMPO (2,2,6,6-tetramethyl-piperidine-N-oxyl)	Nanocellulose with high COOH content	Selective oxidation of primary hydroxyl groups (C6).	[121]
	FF	High-frequency ultrasound 525 to 565 kHz T = 42 °C It uses. H_2_O_2_ **No catalyst**	Maleic acid	Promising route that does not require a catalyst, uses mild temperatures and high-frequency ultrasound	[123]

### 5.4. Sonophotocatalysis, the Emerging Area

Sonophotocatalysis is a new emerging area in obtaining value-added products from lignocellulosic biomass [125,126]. Sonophotocatalysis uses the synergistic effect of ultrasound and light to be more effective in deconstructing lignocellulosic biomass. As an example of a sonophotocatalysis, only the first study of the sonophotocatalytic degradation of lignin with TiO_2_ (photocatalyst) is mentioned, which reached 93% lignin degradation in 180 min using ultraviolet irradiation and ultrasound [127].

## 6. Conclusions

Microwaves (MW) and ultrasound (US) are two emerging techniques for the effective and environmentally friendly processing of lignocellulosic biomass to produce a wide range of value-added bio-based products, from biofuels to different chemicals and advanced bio-based materials. MW- and/or US-assisted processes can provide new biomass conversion routes, thus reducing reaction times, energy consumption and the use of toxic and hazardous solvents, thus fulfilling the basic principles of green chemistry. Given this evidence, it can also be concluded that microwave and ultrasound techniques can greatly contribute to the development of new lignocellulosic biomass conversion processes within the biorefinery and bioeconomy concepts. However, microwaves and ultrasound provide too diverse effects on the lignocellulosic biomass components, affecting their structural integrity and accessibility towards solvents and reagents. In addition to promoting heat and mass transfer, MW and US affect the properties of reaction media, reagents, and catalysts in ways that are often unpredictable. All this requires a very careful, mainly empirical, approach to the selection of specific processing conditions using MW and US for the highly selective conversion of biomass into target products. Thus, there are a series of objective difficulties for the practical application of MW and US in the deconstruction of biomass associated with the complexity of phenomena that occur simultaneously. Some urged challenges and opportunities related to further implementation of MW and US are formulated below.

## 7. Challenges and Perspectives

There are immense challenges in developing new routes for processing lignocellulosic biomass employing emerging microwave and ultrasound techniques, which can be briefly listed as follows:

1. Study the dielectric parameters (dielectric constant, dielectric loss, dielectric loss tangent) of the lignocellulosic biomass concerned before subjecting it to pretreatment with microwave radiation;
2. Understand how the dielectric parameters of lignocellulosic biomass vary with frequency of incident microwaves, and with operating temperature. There are studies that prove that dielectric parameters vary with frequency and temperature, but so far, it seems to be quite difficult to predict the behavior of biomass in the face of these two factors;
3. Develop computational tools to find the best conditions for a given microwave and/or ultrasound pretreatment;
4. Study in more detail the changes in the supramolecular structure of cellulose using microwaves or ultrasound in order to better manage these processes;
5. Advance the study of liquefaction with microwaves on a pilot scale. This is the recommendation of several researchers who argue that they have already been properly tested on a laboratory scale;
6. Expansion of the list of reagents and catalysts for target reactions that use microwaves and/or ultrasound and for reactions that simultaneously use these techniques with other ionizing radiation (for example, ultraviolet light).

## Figures and Tables

**Table 1:** Advantages of microwave heating compared to conventional heating [8,15,25,28,29,30].

Advantage of Microwave Heating	
Non-contact heating	In microwave heating, there is no physical contact between the material to be heated and the heat source. This prevents overheating of the material surfaces.
Lower energy consumption	In conventional heating, the energy consumption is higher since part of the energy is used to heat the container.
Fast heating	In conventional heating, heating is slower.
Lower heat losses	The microwave heating container is non-conductive.
Shorter reaction times	In conventional heating, reaction times are longer.
Volumetric heating	Microwave heating has a uniform heating where the whole material is heated simultaneously, while in conventional heating, there is superficial heating and then a transfer of energy via convection and/or conduction
Better level of control	Microwave heating can be turned off immediately.
Better product yield	In microwave heating, there is low formation of collateral products.
Allows overheating of the material	In microwave heating, the maximum temperature reached is not limited by the boiling point of the substance to be heated.
More selective	Microwave radiation is a more selective than conventional heating because it is only absorbed by the biomass and the reaction media
Improved moisture reduction	In microwave heating, moisture loss occurs first from the surface of the material.

**Table 2:** Classification of materials: in conductors, non-conductors, and dielectrics [8,25,34].

Materials	Characteristics	tanδ	Examples
Conductive	Microwaves cannot penetrate them. They reflect the microwaves.	tan δ<0.1	Metals
Non-conductive	They are microwave-transparent and have low or zero dielectric loss. They are the materials for the construction of containers for microwave heating.	0.1<tan δ<0.5	Glass Teflon Ceramics Quartz Air
Dielectric (or absorbers)	They absorb microwaves. They are ideal to be heated by microwave	tan δ>0.5	Water Methanol Carbon

**Table 3:** Dielectric constants, dielectric losses, dielectric loss tangents, and depths of microwave penetration for various biomasses.

Biomass	$\varepsilon^{\prime }$	$\varepsilon^{\prime \prime }$	tan δ	D_p_ (cm)	Frequencies and Temperature	Reference
Tropical wood	2.08	0.1849	0.0954	----	8.2 to 12.4 GHz	[36]
Banana fibers with polyurethane 30%	137	26	----	----	1 kHz	[37]
Empty fruit bunch (18 wt% moisture)	6.4	1.9	0.3	3.5	2.45 GHz, 27 °C	[35]
Empty fruit bunch char	3.5	0.47	0.13	----	2.45 GHz, 500 °C	[35]
Pinewood	2.7	0.53	----	59	2.45 GHZ, 17 °C	[34]
Oil palm fiber	1.99	0.16	0.08	24.8	2.45 GHZ, 500 °C	[32]
Oil palm shell	2.76	0.35	0.12	13.4	2.45 GHz, 500 °C	[32]
Oil palm char	2.83	0.23	0.08	20.6	2.45 GHz, 500 °C	[32]
Hay	----	----	----	0.02	2.45 GHz, 700 °C	[39]
Pinewood	13.4	0.08	0.006	0.2	2.45 GHZ, 25 °C	[38]
Arabica coffee	26.8	3.14	0.117	0.5	2.45 GHZ, 25 °C	[38]
Wood	----	----	0.11	----	----	[20]
Fir plywood	----	----	0.01–0.05	----	----	[20]
Karanja seeds	----	----	1.3	1.26	0.1 to 3.0 GHz at room temperature	[20]

**Table 4:** Dielectric constant $(\varepsilon^{\prime }$), dielectric loss ($\varepsilon^{\prime \prime }$), and dielectric loss tangent (tan *δ*) for some solvents usually used in the treatments of lignocellulosic biomass (2.45 GHz, at room temperature) [27].

Solvent	$\varepsilon^{\prime }$	$\varepsilon^{\prime \prime }$	**tan** ***δ***
Water	80.4	0.123	9.889
Ethylene glycol	37.0	6.079	0.161
Methanol	32.6	21.483	0.856
Ethanol	24.3	22.866	0.941

**Table 5:** Intra-polymer and inter-polymer bonds in lignocellulosic biomass (adopted from [5]).

Cross Linkages	Types of Bonds	Polymers Involved
Intra-polymer	Ether	Lignin, cellulose, hemicellulose
	Ester	Hemicellulose
	Hydrogen	Cellulose
	C-C	Lignin
Inter-polymer	Ether	Lignin-hemicellulose
	Ester	Lignin-hemicellulose
	Hydrogen	Cellulose-hemicellulose
	Hydrogen	Lignin- cellulose
	Hydrogen	Lignin- hemicellulose

**Table 6:** Some properties of the two categories of ultrasound [27,58,59].

Low- and Medium-Frequency Waves	High-Frequency Waves
20 kHz<f<100 kHz	3 MHz<f<10 MHz
Have high power	Have low power
Suffer cavitation	Do not suffer cavitation
They influence the environment in which they propagate	They do not influence the environment in which they propagate
Applications: Sonochemistry (Part IV- of this review) and industry	Applications: Medical diagnostics and non-destructive control of materials (for example)

**Table 11:** Effect of microwave radiation and ultrasound on the solubilization of organic matter of microalgae biomass [87].

Pretreatment	Operating Conditions	Increase in Soluble Organic Matter	Increase in Soluble Proteins	Increase in Soluble Carbohydrates	Increase in soluble Lipids
MW	P = 900 W f = 2450 MHz ∆t = 3 min	8×	18×	12×	2×
US	P = 70 W f = 20 kHz ∆t = 30 min	7×	12×	9×	3×

**Table 12:** Effect of ultrasound on the increase of sCOD efficiency (%) in two species of microalgae [88].

Biomass	Pretreatment	Operating Conditions	Increased sCOD ^1^ Efficiency (%)
*Tetraselmis suecica*	US	P = 500 W f = 20 kHz ∆t = 5 s T = 19.1 °C	5.13
*Nannochloropsis oceanica*	US	P = 500 W f = 20 kHz ∆t = 54 s T = 21.6 °C	18

^1^ sCOD—soluble chemical oxygen demand.

## Data Availability

Not applicable.
